# Physical Therapy Management in a Severe Case of Overlapping of Bone Post Crush Injury: A Case Report

**DOI:** 10.7759/cureus.29708

**Published:** 2022-09-28

**Authors:** Chaitanya A Kulkarni, Om C Wadhokar, Medhavi V Joshi

**Affiliations:** 1 Community Health Physiotherapy, Ravi Nair Physiotherapy College, Datta Meghe Institute of Medical Sciences, Wardha, IND; 2 Musculoskeletal Physiotherapy, Ravi Nair Physiotherapy College, Datta Meghe Institute of Medical Sciences, Wardha, IND

**Keywords:** dislocation, fracture, thumb carpometacarpal joint, physiotherapy rehabilitation, crush injury

## Abstract

Crush injuries are sustained due to high velocity and usually have a poor outcome. Since the compressive forces are of high energy, such injuries are usually seen in road traffic accidents or in industrial workers. Crush injuries of the hand account for relatively a smaller percentage of the injuries to the hand and include an open wound along with fracture dislocation of the carpometacarpal, interphalangeal, and radiocarpal joints. Since these injuries are uncommon, they are often overlooked in radiological findings or misdiagnosed. Their recognition depends on a careful physical and radiographic examination that may require trispiral computed tomograms. Physical therapy rehabilitation post-surgery becomes a necessity, especially in cases where the dominant hand is affected. Functional rehabilitation to improve the independence and efficiency of activities of daily living is a goal of utmost importance.

## Introduction

The carpometacarpal joints are a type of plane synovial joints that provide articulation between the carpals and the metacarpals, except for the first carpometacarpal joint which is a saddle joint [1]. The dislocation of these joints of fingers is rare and accounts for less than one percent of hand injuries, with predominance of occurrence in males, as it is usually caused due to high-velocity trauma [2]. The two types of injury are volar and dorsal, which depend upon the direction of displacement. The dorsal displacement in the injury is common due to strong restraints from wrist extensors and static dorsal ligaments. Carpometacarpal joint fracture dislocations are managed surgically with either closed or open reduction [3]. The prognosis is comparatively better when treated with open reduction as the chances of re-dislocation are reduced [4].

The physical therapy rehabilitation begins after six weeks of immobilization for the affected hand and wrist. For the unaffected extremity, physical therapy begins post-operative day one to improve functional independence, as the dominant hand is usually involved [5]

## Case presentation

A left-hand-dominant 38-year-old male with a desk job and high screen time presented to the orthopaedic department with a history of road traffic accident nine hours ago, while driving a four-wheeled vehicle. Due to the presence of an open wound on his left hand, he was immediately taken to the nearby hospital and self-management by wrapping up of wound with a cloth to prevent excessive blood loss was done. Further dressing and referral to a multispecialty hospital were done. He had no pathological precursor or any history of previous surgery or trauma.

**Figure 1:**
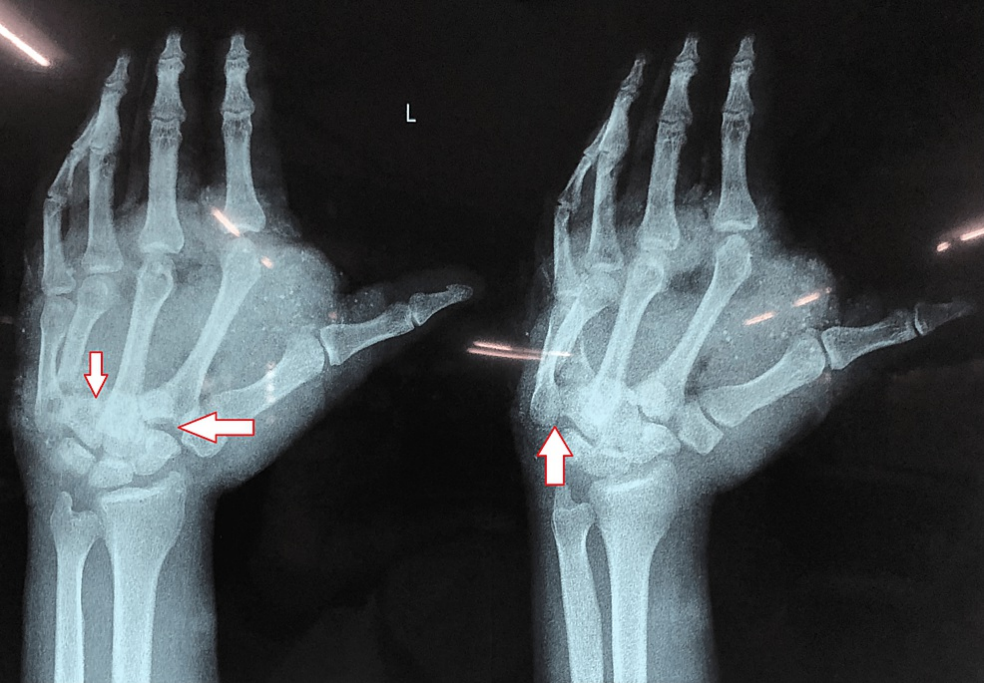
Pre-operative anteroposterior view of X-ray The image on the left shows left-hand fracture dislocation of the second, third, fourth, and fifth carpometacarpal joints. The image on the right indicated fracture of the base of fifth metacarpal.

The patient gave a history of immediate onset of pain after the accident and was aggravated by movements with diffuse swelling present over the hand (dorsal and palmar aspect). Tendons were exposed at the dorsal aspect of the hand. A lacerated wound of 8 x 10 cm over the palmar aspect of the hand extending from the thenar eminence and a 10 x 10 cm lacerated wound over the dorsal aspect of the hand were present. He was then investigated with an X-ray which revealed fracture dislocation of the second, third, fourth, and fifth carpometacarpal joints with fracture of the base fifth metacarpal (Figure 1).

**Figure 2:**
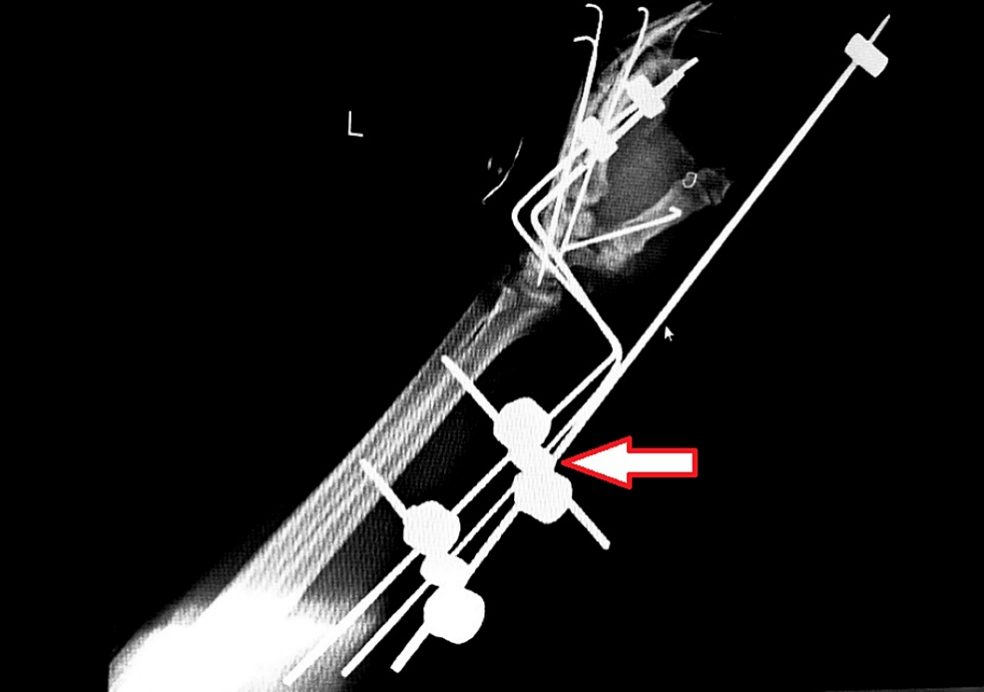
Post-operative X-ray of the left hand The implant seen is a JESS ( Joshi's External Stabilization system) fixator

**Figure 3:**
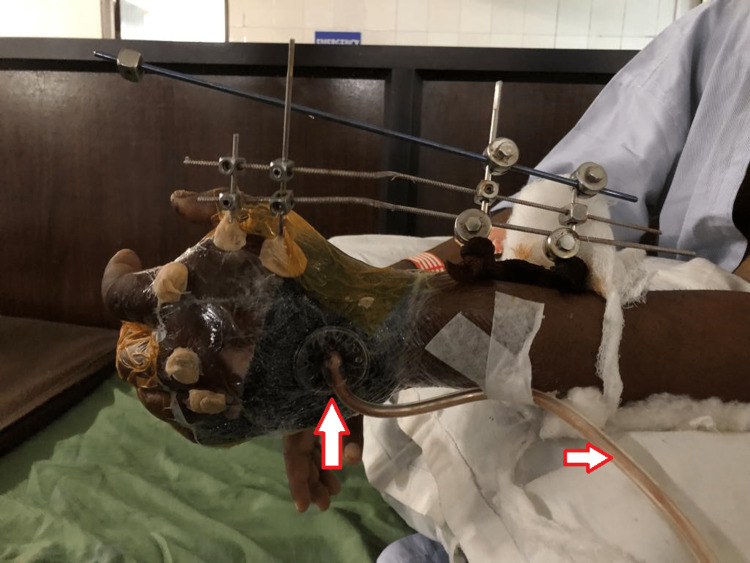
Vacuum-assisted closure (VAC) Vacuum-assisted closure (VAC) is present to facilitate healing. The translucent tube is present to generate a negative pressure which prevents surgical site infection, a common complication when managed with external fixator.

Surgery was performed, where intraoperatively, it was revealed that a contused lacerated wound over the palmar and dorsal aspect of the left hand extending from the base of the thumb across the palm was present with severe crushing of tissues on both sides. Hypothenar muscles were necrosed and gross contamination was present with soil and grass. Debridement of the wound and open reduction and internal fixation with Kirschner wire (K-wire) of the second, third, fourth, and fifth carpometacarpal joints, base of fifth metacarpal was grossly comminuted so K-wire was not passed through it. Reduction and fixation conform under C-arm. Extensor tendons were intact. Cock up slab was given over left upper limb. After a week, the K-wire was removed and JESS (Joshi's External Stabilization System) external fixator was applied (Figure 2 and Figure 3).

Physical therapy assessment and rehabilitation

Clinical Findings

On observation, the patient was lying in a supine position with the affected hand flexed to 90 degrees at the elbow. Proximal and distal interphalangeal joints were in 5-10 degrees of flexion. The Pulse could not be palpated due to the presence of the external fixator and dressing. On examination, the pain was assessed on the Numerical Pain Rating Scale and was during movement 8/10 and at rest 2/10. Range of motion assessed with a goniometer for all the ranges of the left upper limb at shoulder and elbow were with normal functional ranges. Ranges of wrist and all the joints of the hand could not be assessed due to pain, dressing, and the presence of external fixator.

Timeline 

The patient was subjected to injury in the last week of January. He was bought to the orthopaedic department nine hours later from the time of the accident on January 27, 2022. He was operated on January 28, 2022. Physical therapy rehabilitation started from January 29, 2022 and continued till February 7, 2022, in hospital and further five days of telerehabilitation till February 12, 2022.

Physiotherapeutic Intervention

Physiotherapy rehabilitation began post-operatively from day one, when the patient and his caregivers were educated about the importance of exercise therapy for early recovery, to maintain integrity of surrounding musculature and soft tissues, to prevent post-operative stiffness and flexion deformity, and to maintain and strengthen the unaffected muscles. Day one to day three of physical therapy included active range of motion exercises for elbow flexion, shoulder flexion (0-180 degrees), and abduction (0-90 degrees) with the elbow flexed at 90 degrees. A total of 30-40 repetitions/day for three days was done. Day four to day seven included all the previous exercises with an increased number of repetitions, that is, 50-70 repetitions/day. Shoulder abduction with complete elbow extension and cross-body adduction to were added. Mild active finger movements were also started. Donning and doffing of the clothes was also taught to the patient to improve the independency level.

Functional activities performed with the right hand were started, as the patient was left-hand dominated. To facilitate independent eating activities, the activities started were crushing a paper to improve intrinsic muscle strength, tearing of the paper with the thumb, index and middle fingers (the Indian method of eating with hand involves this action), and holding a spoon and bringing it towards the mouth and back.

Days 8-12 included all the previous activities along with strengthening exercises for unaffected right upper extremity and affected side shoulder. Drawing using the non-dominant hand for 20 mins and typing 100 words every day were added [6]. Telerehabilitation was done twice a week post-discharge, where the patient was observed for any trick movements and modifications needed in the household as per the work requirements.

Patient’s Perspective

Initially, the patient was reluctant to perform exercises, but as he could see a reduced level of pain and stiffness, he started complying with the treatment protocol.

## Discussion

We have reported a case of fracture dislocation of the second, third, fourth, and fifth carpometacarpal joint with the base of the fifth metacarpal fracture. We report this case as the occurrence of crush injuries accounts for a very small percentage of injuries to the hand, and therefore we highlight its diagnosis and surgical intervention along with rehabilitation post-surgically. A study reports difficulty in adduction of the second and fifth fingers due to palmar interosseous muscle weakness, carpal deformity at the fourth carpometacarpal joint, and damage to the digital nerve leading to loss of sensations and non-union as a few complications [7]. As a part of rehabilitation in the acute phase, educating the patient regarding the same complications is of importance for early detection and management. For physical therapy rehabilitation, a study reports that shorter periods of immobilization should be followed by isolated and combined motions of metacarpophalangeal and interphalangeal joints and flexion and extension of the radiocarpal joints [8]. Physiotherapy rehabilitation in the acute stage was aimed at maintaining the integrity of all the joints proximal and distal to the location of the injury and to effectively improve the efficiency in performing activities with the non-dominant hand. Crush injuries usually involve multiple fractures and dislocation and take time to heal and therefore usually account for chronic disability in affected individuals [9]. A need, therefore, is to minimize this disability through conditioning the patient to various activities that would otherwise be difficult to perform if not trained [10].

## Conclusions

Diligence in diagnosis will help prevent missing out on small but significant injuries during diagnosis of crush injuries of the hand. After diagnosis, prognosis then depends upon early reduction, stabilization, and rehabilitation. Early rehabilitation should include optimizing the functioning of the patient using non-dominant extremity as it also has a positive psychological impact on the patient. The treatment protocol is individualized depending upon the severity of the injury and needs a long-term follow-up and vigorous physiotherapy to attain functional ranges and strength.
